# Organisational ethical climate and nurses’ work performance: A cross-sectional analysis of the indirect association through job crafting

**DOI:** 10.1177/10519815261473193

**Published:** 2026-08-05

**Authors:** Mehmet Gülşen, Nurşah Tatoğlu, Ayşe Karadaş

**Affiliations:** 1 Department of Medical Services and Techniques, İvrindi Vocational School of Health Services, 53003 Balıkesir University, Balıkesir, Türkiye; 2 Department of Nursing, Faculty of Health Sciences, 53003 Balıkesir University, Balıkesir, Türkiye

**Keywords:** hospitals, nursing, occupational stress, personnel management, professional competence, work engagement, workplace

## Abstract

**Background:**

Organisational ethical climate is relevant to nursing practice and employee outcomes, but its association with nurses’ work performance has received limited attention, particularly in relation to job crafting.

**Objective:**

To examine the associations between organisational ethical climate, job crafting, and work performance among nurses in Türkiye, and to assess whether job crafting statistically mediated the association between ethical climate and work performance.

**Methods:**

This descriptive cross-sectional study included 204 nurses from a university hospital in Türkiye. Data were collected between June and September 2024 using the Organisational Ethical Climate Scale, the Job Crafting Scale, and the Individual Work Performance Questionnaire. Analyses were performed using IBM SPSS Statistics 26 and the PROCESS macro v4.2.

**Results:**

Organisational ethical climate was positively correlated with job crafting (r = .359, p < .001) and work performance (r = .346, p < .001). Job crafting was also positively correlated with work performance (r = .503, p < .001). The indirect effect of ethical climate on work performance through job crafting was significant (ab = .096, 95% CI [.042, .176]). The direct effect remained significant (c′ = .130, p = .004), indicating partial statistical mediation.

**Conclusions:**

A more positive ethical climate was associated with higher job crafting and better self-reported work performance. Job crafting statistically accounted for part of the association between ethical climate and work performance.

## Introduction

Nurses are expected to deliver safe and high-quality care in work environments characterised by high workloads, time pressure, and organisational constraints.^1^ Empirical evidence indicates that healthier workplace conditions are associated with higher perceived quality of care and safer patient outcomes.^2^ Consequently, identifying organisational and behavioural mechanisms that can enhance nurses’ performance in resource-constrained environments has become a central concern in contemporary nursing management research.

One contextual factor that has received increasing attention in this regard is ethical climate. This construct refers to shared perceptions of what constitutes ethically appropriate behaviour within an organisation and how ethical issues should be handled.^3^ Ethical climate therefore represents a salient organisational feature that can influence how nurses interpret, enact, and sustain proactive work behaviours. In nursing settings, ethical climate has been linked to job satisfaction,^4^ moral distress, and turnover intention,^5^ all of which are closely related to nurses’ work performance. Consistent with this view, Park and Jeong^6^ found that hospital ethical climate was positively associated with nursing task performance, indicating that nurses working in ethically supportive environments perform their professional roles more effectively. Supportive ethical climates are associated with greater psychological safety and clarity about acceptable conduct, whereas ethically strained environments are associated with uncertainty, stress, and reduced effectiveness at work. Such organisational conditions affect not only how nurses feel, but also how they organise their work and adjust their daily practices.^7^ These daily adjustments draw attention to job crafting as a proactive behavioural mechanism through which nurses may translate a supportive ethical climate into more effective work practices.

Job crafting refers to employees’ proactive efforts to modify the task, relational, and cognitive boundaries of their work in order to achieve a better alignment between their jobs and their personal strengths, values, and needs.^8^ From a Job Demands–Resources perspective, these changes involve adjusting job demands, which are aspects of work that require sustained effort and entail psychological or physical costs, and job resources, which are aspects of work that support goal attainment, reduce strain, and promote learning and development. Through job crafting, employees can therefore increase their resources and regulate their demands, thereby actively shaping the motivational and strain-related qualities of their jobs.^9^

In demanding work environments, nurses’ capacity to adapt how they organise tasks and coordinate care becomes increasingly important for maintaining work performance. A growing body of research indicates that when nurses actively shape how they carry out their work, they tend to be more engaged^10^ and to perform more effectively.^11^ At the same time, the extent to which such proactive behaviours translate into performance gains may depend on characteristics of the organisational context.

In this regard, ethical climate may represent a key contextual factor that supports or constrains job crafting. When nurses perceive their organisation as fair, supportive, and ethically consistent, they may be more willing to take initiative, seek support, and adjust their work practices in ways that improve fit between job demands and available resources. By contrast, in ethically strained environments, concerns about blame, conflict, or unfair treatment may limit nurses’ willingness to engage in such proactive changes.^12^

Previous research has consistently linked organisational ethical climate to nurses’ attitudes, such as job satisfaction, moral distress, and turnover intention,^4,5^ but much less is known about how it relates to their actual work behaviours and performance. Studies on job crafting in nursing have mostly focused on the work environment and practice setting,^13,14^ and have rarely considered ethical climate. Moreover, these three variables have seldom been examined together in a single model. It therefore remains unclear whether job crafting helps explain the link between ethical climate and performance, or whether this link is also shaped by other organisational or motivational factors. Accordingly, examining the behavioural foundations of the relationship between ethical climate and work performance represents an important research need. In this context, examining ethical climate alongside the proactive behaviours through which nurses adapt their work has the potential to address an important gap in the literature. Therefore, the present study aimed to examine the associations between organisational ethical climate, job crafting, and nurses’ work performance in a Turkish university hospital, and to explore whether job crafting statistically accounted for part of the association between organisational ethical climate and work performance.

### Hypotheses

**H1.** Organisational ethical climate is positively associated with nurses’ job crafting.
**H2.** Nurses’ job crafting is positively associated with their work performance.
**H3.** Organisational ethical climate is positively associated with nurses’ work performance.
**H4.** Organisational ethical climate has a statistically significant indirect association with nurses’ work performance through job crafting.

## Methods

### Study design and sample

This study was conducted using a descriptive cross-sectional design at a university hospital. The study population consisted of all nurses working in the hospital during the data collection period. A convenience sample of eligible nurses was recruited from various clinical units within the hospital. Before data collection, the minimum sample size required for the mediation analysis was estimated in line with Fritz and MacKinnon's recommendations.^15^ Based on anticipated small-to-moderate effects for both the a and b paths (approximately .26), the required sample size for the percentile bootstrap test was 162. To account for potential non-response or unusable questionnaires, this minimum sample size was increased by 20%, resulting in a recruitment target of at least 195 participants. A total of 300 nurses working in a university hospital were invited to participate. Of these, 32 declined to participate, primarily because of a heavy workload, 51 were unavailable during the data collection period (e.g., because of medical or annual leave), and 13 returned incomplete questionnaires, which were excluded from the analyses. The final sample comprised 204 nurses who provided informed consent and met the inclusion criteria (response rate = 68.0%). Thus, the final analysed sample exceeded the planned minimum target. Inclusion criteria were: (1) age ≥18 years and (2) current employment as a nurse.

### Data collection instruments

Data were collected using a Descriptive Information Form, the Organisational Ethical Climate Scale, the Individual Work Performance Questionnaire, and the Job Crafting Scale.

### Descriptive information form

This form comprised nine items assessing sociodemographic and occupational characteristics: age, gender, educational level, marital status, professional experience, institutional tenure, unit of employment, position, and weekly working hours.

### Organisational ethical climate scale

The Organisational Ethical Climate Scale was developed by Çalışkan.^16^ It is a five-point Likert-type scale that measures organisational ethical climate through two dimensions, namely the climate of rules and the climate of actions. For the present study, an overall mean score was calculated across all items, and this overall score was used in the mediation analyses. The overall score was selected to align with the construct-level focus of the proposed mediation model. Higher scores indicate a more positive organisational ethical climate. The original study reported a Cronbach's alpha of .89^16^; in the present study, Cronbach's alpha was .95.

### Individual work performance questionnaire

This scale was developed by Koopmans et al.^17^ and was cross-culturally validated.^18^ The Turkish version was adapted by Kaba and Öztürk.^19^ It consists of 14 items across three subscales: task performance, contextual performance, and counterproductive work behaviour. Items are rated on a five-point Likert scale ranging from 1 to 5. Items related to counterproductive work behaviour were reverse coded before calculating the overall performance score. After reverse coding, the item scores were averaged to produce an overall work performance score. Accordingly, higher overall scores indicate better perceived work performance. In the Turkish adaptation study, Cronbach's alpha was reported as .80,^19^ whereas Cronbach's alpha was .87 in the present study.

### Job crafting scale

The Job Crafting Scale, developed by Tims et al.,^20^ was used to measure employees’ job crafting behaviours. The Turkish validity and reliability study was conducted by Çetin et al.^21^ The scale consists of 21 items across four subscales: (1) increasing structural job resources, (2) decreasing hindering job demands, (3) increasing social job resources, and (4) increasing challenging job demands. Items are rated on a five-point Likert scale (1 = never, 5 = often). For the present study, an overall mean score was calculated across all items, and this overall score was used in the mediation analyses. Higher scores indicate higher levels of job crafting. Cronbach's alpha was reported as .86 in the Turkish adaptation study^21^ and was .86 in the present study.

### Data collection procedure

Following approval from the ethics committee and the relevant institutional authorities, data were collected between June and September 2024. Data collection was conducted face-to-face by the researchers during institution-approved time periods, in a manner that did not disrupt routine clinical operations. Eligible nurses were informed about the study by the researchers in person and invited to participate on a voluntary basis. Participants completed the questionnaires individually; the average completion time was approximately 15 min. Researchers remained available to clarify procedural questions (e.g., how to complete the forms) but did not influence participants’ responses. This individual and non-intrusive administration procedure was intended to enhance response quality and reduce potential same-source bias.

### Data analysis

Analyses were performed using IBM SPSS Statistics 26 and the PROCESS macro v4.2 plug-in.^22^ Statistical significance was set at p < .05 (two-tailed). Univariate normality was assessed using skewness and kurtosis z-values (|z| < 1.96).^23^ Multivariate outliers were screened using Mahalanobis distance with a p < .001 criterion. The assumptions of regression analysis, including linearity, homoscedasticity, and multicollinearity, were assessed, and no substantial violations were detected. Descriptive statistics (frequencies, percentages, means, and standard deviations) were computed, and internal consistency was assessed using Cronbach's alpha. Pearson correlations were calculated and interpreted using the criteria proposed by Schober et al.^24^ There were no missing data for the study variables; therefore, all analyses were conducted on complete cases (n = 204). Preliminary analyses were performed to assess whether selected sociodemographic and occupational characteristics were associated with the main study variables. The covariates were selected on both conceptual and empirical grounds. Gender, educational level, unit of employment, and marital status have been associated in previous nursing research with perceptions of the work environment, ethical climate, and proactive work behaviours, and were therefore considered potentially relevant to the focal constructs. To verify their relevance in the present sample, preliminary analyses examined their associations with the study variables, and variables showing significant associations were retained as covariates. Unit of employment and educational level were entered using dummy coding. Indirect effects were tested using Hayes’ PROCESS macro (Model 4) with 5000 bootstrap resamples, and statistical significance was determined based on 95% percentile bootstrap confidence intervals. As a supplementary post hoc analysis, a Monte Carlo simulation for a simple mediation model was conducted using the achieved sample size (n = 204). The estimated power to detect the indirect effect exceeded .99, indicating adequate sensitivity of the achieved sample. To assess potential common method variance, Harman's single-factor test was conducted by performing an unrotated exploratory factor analysis on all measurement items.^25^ The first unrotated factor explained 25.10% of the total variance, suggesting that no single factor accounted for the majority of variance in the measures.

### Ethical considerations

Ethical approval was obtained from the Non-Interventional Ethics Committee of Balıkesir University (Approval No: 2024/64). Institutional approval was obtained from the hospital, and written permission was secured from the scale developers. All participants provided informed consent prior to participation. The study was conducted in accordance with the principles of the Declaration of Helsinki.

## Results

Table 1 presents the characteristics of the participants. The mean age was 35.75 years, with 62.3% aged 40 years or younger. Females comprised 85.3% of the sample. A total of 53.4% held a bachelor's degree and 11.3% had postgraduate education. A total of 61.8% of the participants were married.

**Table 1.** Characteristics of the participants (n = 204).

Variables	n	%
*Age*		
≤ 40 years	127	62.3
> 40 years	77	37.7
*Gender*		
Male	30	14.7
Female	174	85.3
*Educational level*		
High school	29	14.2
Associate degree	43	21.1
Bachelor's degree	109	53.4
Master's or PhD	23	11.3
*Marital status*		
Married	126	61.8
Single	78	38.2
*Professional experience*		
1–10 years	90	44.1
11–20 years	58	28.4
21–30 years	41	20.1
> 30 years	15	7.4
*Institutional experience*		
1–5 years	95	46.6
6–10 years	88	43.1
11–15 years	21	10.3
*Unit of employment*		
Inpatient clinic	95	46.6
Emergency department	23	11.3
Intensive care	56	27.5
Outpatient clinic	18	8.8
Operating room	12	5.9
*Position*		
Nurse	181	88.7
Nurse manager	23	11.3
*Weekly working hours*		
40–56 h	131	64.2
57–96 h	73	35.8

Regarding experience, 44.1% had 1–10 years of professional experience, and 46.6% had worked in the institution for 1–5 years. In terms of work setting, 46.6% worked in inpatient clinics and 27.5% in intensive care units. Most participants were staff nurses (88.7%). In terms of weekly working hours, 64.2% worked 40–56 h per week, whereas 35.8% worked 57 h or more.

Table 2 presents the means, standard deviations, and bivariate correlations among organisational ethical climate, job crafting, and work performance. The mean score for organisational ethical climate was 3.22 (SD = 1.00). Job crafting had a mean of 3.65 (SD = .52), and work performance had a mean of 3.77 (SD = .63). Organisational ethical climate was positively correlated with job crafting (r = .359, p < .001) and with work performance (r = .346, p < .001). Job crafting was also positively correlated with work performance (r = .503, p < .001). These results provide initial support for H1, H2, and H3.

**Table 2.** Descriptive statistics and correlations among variables.

Variables	Mean	SD	Range	1	2	3
1. Organisational ethical climate	3.22	1.00	1–5	—		
2. Job crafting	3.65	.52	1–5	.359***	—	
3. Work performance	3.77	.63	1–5	.346***	.503***	—

*Note:* Values are Pearson correlation coefficients. Scale scores represent overall mean item scores on the original 1–5 metric. n = 204. ***p < .001.

Preliminary analyses were conducted to examine associations between participants’ sociodemographic and occupational characteristics and the main study variables. No significant associations were found between work performance and any sociodemographic or professional variables (p > .05). Job crafting scores were significantly associated with marital status (t = 3.156, p = .002; single > married) and unit of employment (Kruskal–Wallis χ^2^ = 14.141, p = .007). Organisational ethical climate scores were significantly associated with gender (t = 2.152, p = .033; males > females), educational level (χ^2^ = 14.710, p = .002), and unit of employment (Kruskal–Wallis χ^2^ = 16.859, p = .002). Based on these findings, marital status, gender, educational level, and unit of employment were included as covariates in the mediation model.

The mediation analysis demonstrated a statistically significant indirect effect of organisational ethical climate on work performance via job crafting (ab = .096, 95% bootstrap CI [.042, .176]). Organisational ethical climate was positively associated with job crafting (a = .181, p < .001), and job crafting was, in turn, positively associated with work performance (b = .529, p < .001). The direct effect of ethical climate on work performance remained statistically significant (c′ = .130, p = .004), indicating partial statistical mediation (Table 3). As illustrated in Figure 1, these findings support H1, H2, H3, and H4. Each one-point increase in organisational ethical climate was associated with a .225-point increase in work performance. Of this total association, an estimated .096 points were attributable to the indirect path through job crafting.

**Figure 1.**
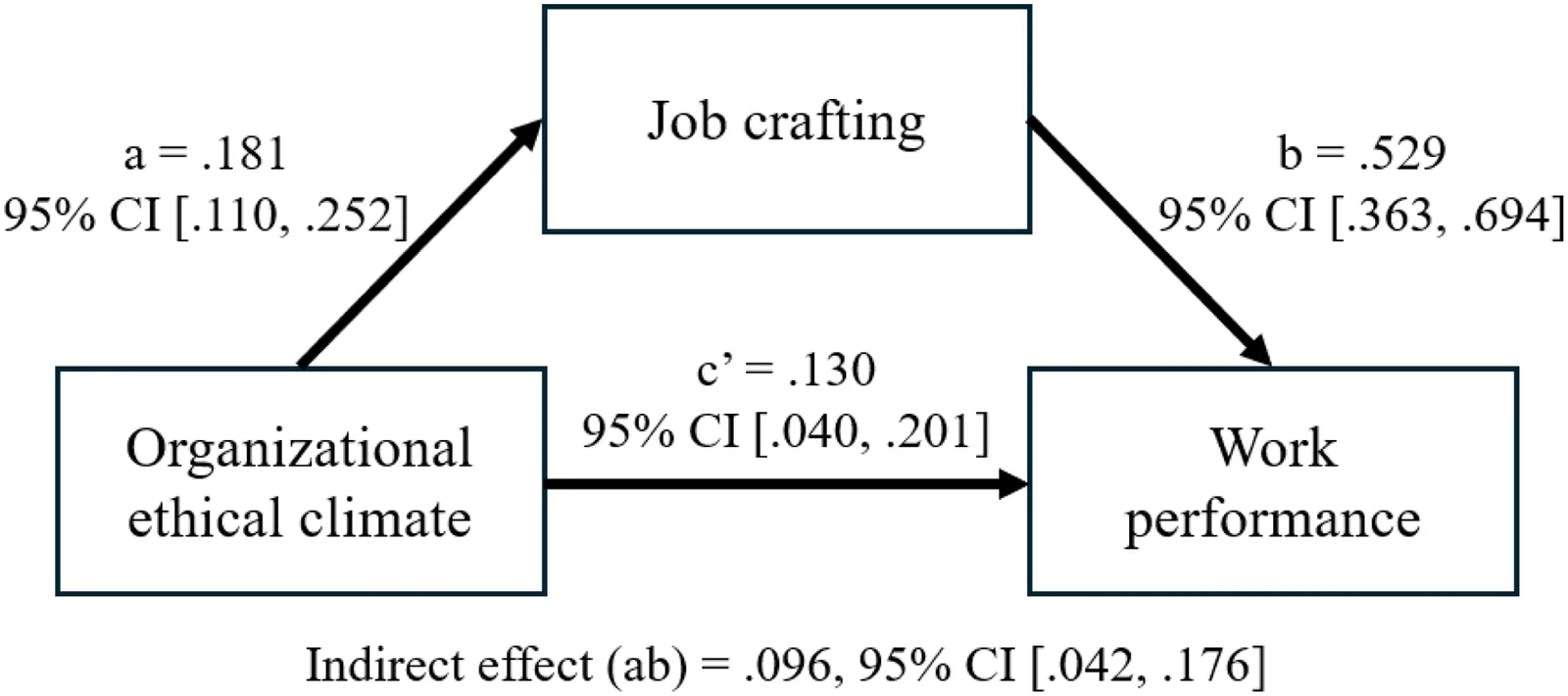
Mediation model of job crafting in the relationship between organisational ethical climate and work performance.

**Table 3.** Mediation analysis results.

Path	B	SE	t	p	95% CI
Indirect effect					
Ethical Climate → Job Crafting → Work Performance	.096	.035	—	—	[.042, .176]
Direct effects					
Ethical Climate → Job Crafting (a)	.181	.036	5.047	<.001	[.110, .252]
Job Crafting → Work Performance (b)	.529	.084	6.310	<.001	[.363, .694]
Ethical Climate → Work Performance (c′)	.130	.044	2.920	.004	[.040, .201]
Total effect					
Ethical Climate → Work Performance (c)	.225	.046	4.926	<.001	[.135, .315]

*Note:* B = unstandardised regression coefficient; SE = standard error; t = t value; p = significance level; CI = confidence interval; a = effect of ethical climate on job crafting; b = effect of job crafting on work performance; c′ = direct effect of ethical climate on work performance controlling for job crafting; c = total effect of ethical climate on work performance.

## Discussion

Sustaining high work performance in nursing requires attention to organisational conditions that shape how nurses interpret and enact their roles, as well as the proactive behaviours through which they adapt their work under high demands. In this context, the present study examined the associations among organisational ethical climate, job crafting, and nurses’ work performance, and explored whether job crafting statistically accounted for part of the association between organisational ethical climate and work performance.

The results of this study showed a significant and positive relationship between organisational ethical climate and job crafting among nurses, supporting H1. Although empirical studies that examine ethical climate and job crafting together in nursing appear to be scarce, research using closely related contextual constructs suggests a similar pattern. In nursing settings, job crafting has been linked to organisational culture and organisational health,^26^ the nursing work environment,^13^ and the nursing practice environment.^14^ It has also been examined in relation to patient safety culture^27^ and broader psychosocial and social climate conditions relevant to nurses’ work experiences.^28,29^ These findings collectively suggest that supportive organisational conditions may coincide with greater proactive work adjustment among nurses.

Job crafting was positively related to nurses’ work performance, supporting H2. This finding is consistent with prior nursing research linking job crafting to nurse performance, including Bacaksiz et al.,^30^ who reported a comparable correlation (r = .51, p < .001), and Ryu,^31^ who found both a positive association (r = .42, p < .001) and a significant predictive effect (β = .20, p < .001). Similar evidence has also been reported for related care outcomes, including quality of nursing services^32^ and person-centred care.^14^ At the same time, evidence from missed nursing care suggests that the effects of specific job crafting dimensions may vary across roles and contexts.^33^ Accordingly, the present findings support the general relevance of job crafting to nurses’ work performance, while also indicating that its implications may depend on the form of job crafting and the clinical context.

Organisational ethical climate was positively associated with nurses’ work performance in the mediation model, and the total effect was also significant, supporting H3. This finding is consistent with previous studies linking ethical climate to nursing performance^6,34^ and in-role and extra-role service behaviours.^35^ For example, Park and Jeong^6^ reported that hospital ethical climate was positively associated with nursing task performance (β = .46, p < .001), whereas Çalışkan et al.^34^ found positive paths from ethical climate to contextual performance (β = .55, p < .001) and task performance (β = .10, p < .001). In addition, review evidence suggests that ethical climate is relevant to organisational outcomes such as turnover intention, operating through work-related attitudes including commitment and job satisfaction. This further underscores its relevance to nursing outcomes and organisational functioning.^36^ Taken together, these findings suggest that ethical climate may be relevant not only to how nurses perceive the moral quality of their work environment, but also to how effectively they perform within it.

The results further indicated that job crafting statistically accounted for part of the association between organisational ethical climate and nurses’ work performance, supporting H4. Although studies testing ethical climate, job crafting, and performance within a single model appear to be limited, job crafting has been reported as a mediator between strengths use and turnover intention,^37^ sparking leadership and work engagement through team job crafting,^38^ and positive psychological capital and work engagement.^39^ Evidence from broader occupational settings has also supported the mediating role of job crafting in the relationship between work engagement and job performance.^40^ In the present study, job crafting partly accounted for the association between ethical climate and work performance, suggesting that additional organisational factors, such as leadership support, staffing adequacy, or work engagement, may also contribute to this relationship.

The findings should be interpreted within the organisational and cultural context of the participating Turkish university hospital. Ethical climate perceptions, opportunities for job crafting, and performance expectations may vary across institutional structures, managerial practices, and healthcare systems. Therefore, the generalisability of the findings is likely to be greatest in similar university hospital settings and should be considered more cautiously in other organisational or cultural contexts.

### Implications for practice

The findings suggest several considerations for practice within the organisational context studied. First, organisational ethical climate should be understood not merely as an abstract ethical ideal, but as a modifiable contextual factor that may be relevant to nurses’ work performance. Nurse managers can strengthen ethical climate by clarifying ethical expectations, ensuring fairness and consistency in decision-making, and maintaining psychologically safe channels through which ethical concerns can be raised without fear of blame or retaliation. Second, because job crafting partially accounted for the association between ethical climate and performance in this study, nurse managers may consider supporting appropriate job crafting behaviours among nurses. This may involve providing structured opportunities for nurses to reflect on workload-related challenges, encouraging peer consultation, and demonstrating managerial openness to adjusting task allocation and care coordination practices within professional and patient safety boundaries, particularly in high-demand work settings. More concretely, nurse managers may support constructive job crafting by incorporating brief reflective discussions into routine shift huddles, encouraging minor workflow adjustments that do not alter core clinical responsibilities, and facilitating peer-based problem solving during high-demand periods. Any such adaptations should remain consistent with clinical protocols, patient safety requirements, and organisational standards. These implications may be more applicable to comparable university hospitals or similarly structured institutional environments than to other settings. Finally, the observed associations suggest that the potential benefits linked to job crafting may be more evident when accompanied by a supportive ethical climate and additional organisational resources.

### Limitations

This study contributes cross-sectional evidence on the associations between organisational ethical climate, job crafting, and nurses’ work performance. However, several limitations should be acknowledged. Given the cross-sectional design and the collection of all variables through self-report questionnaires at a single time point, the indirect effect should be interpreted as statistical rather than causal mediation, and the findings may be affected by common method variance and social desirability or self-enhancement bias. In addition, the use of overall scale scores may have obscured dimension-specific associations. Future studies should consider supervisor-rated or objective performance indicators and dimension-level analyses. The study was conducted in a single university hospital using convenience sampling, which may limit the representativeness of the sample and the generalisability of the findings. Because ethical climate perceptions, opportunities for job crafting, and performance expectations may vary across hospital types, managerial structures, and healthcare contexts, the findings should be interpreted as specific to the organisational setting studied and generalised cautiously beyond similar institutional environments. In addition, the covariates were identified partly through their statistical associations with the study variables, an approach that may capture sample-specific patterns and may not account for all relevant confounders. Future research using larger and more diverse samples, longitudinal or multi-wave designs, and multi-source performance indicators would strengthen external validity and help clarify the temporal structure of these associations.

## Conclusions

The findings align with prior work showing positive links among organisational ethical climate, proactive work behaviours, and performance in nursing. In the present study, organisational ethical climate was positively associated with nurses’ work performance, and job crafting statistically accounted for part of this association. Within the participating university hospital, these findings suggest that ethically supportive work environments may coincide with stronger self-reported performance and more proactive work adaptation among nurses. These findings highlight the value for nurse managers of strengthening ethical climate while also supporting appropriate job crafting practices within professional boundaries.
